# Formation of electron radiation belts at Saturn by Z-mode wave acceleration

**DOI:** 10.1038/s41467-018-07549-4

**Published:** 2018-11-29

**Authors:** E. E. Woodfield, R. B. Horne, S. A. Glauert, J. D. Menietti, Y. Y. Shprits, W. S. Kurth

**Affiliations:** 1 British Antarctic Survey, High Cross, Madingley Road, Cambridge, CB3 0ET UK; 2 Department of Physics and Astronomy, University of Iowa, Iowa City, IA 52242 USA; 3 Helmholtz Centre Potsdam, GFZ German Research Centre for Geosciences, Potsdam, 14473 Germany; 4 Institute for Physics and Astronomy, Universität Potsdam, 14469 Potsdam, Germany; 5 Department of Earth, Planetary, and Space Sciences, University of California, Los Angeles, CA 90095 USA

## Abstract

At Saturn electrons are trapped in the planet’s magnetic field and accelerated to relativistic energies to form the radiation belts, but how this dramatic increase in electron energy occurs is still unknown. Until now the mechanism of radial diffusion has been assumed but we show here that in-situ acceleration through wave particle interactions, which initial studies dismissed as ineffectual at Saturn, is in fact a vital part of the energetic particle dynamics there. We present evidence from numerical simulations based on Cassini spacecraft data that a particular plasma wave, known as Z-mode, accelerates electrons to MeV energies inside 4 R_S_ (1 R_S_ = 60,330 km) through a Doppler shifted cyclotron resonant interaction. Our results show that the Z-mode waves observed are not oblique as previously assumed and are much better accelerators than O-mode waves, resulting in an electron energy spectrum that closely approaches observed values without any transport effects included.

## Abstract (web summary)

Radial diffusion is the only mechanism considered to accelerate trapped electrons to relativistic energies in Saturn’s magnetic field, forming radiation belts. Here the authors show another mechanism, electron acceleration via Doppler shifted cyclotron resonant interaction with Z-mode waves, which can form radiation belts inside the orbit of Enceladus.

## Introduction

Radiation belts are formed when charged particles (usually electrons and protons) are trapped by large scale planetary magnetic fields. The particles then undergo significant acceleration up to relativistic energies^1–3^. Radial diffusion is usually assumed to be the dominant mechanism accelerating electrons in planetary radiation belts^4^. In recent years, however, acceleration by Doppler shifted cyclotron resonant wave particle interactions has been shown to be a key process in the Earth’s radiation belts^5–8^ and at Jupiter^9,10^. However, at Saturn, local wave particle interactions have been dismissed as unlikely to significantly accelerate electrons^11,12^.

Whilst radial diffusion can result in transport of charged particles towards the high magnetic field strength nearer the planet thus increasing their energy (assuming the first two adiabatic invariants are conserved), wave particle interactions are a local process acting in situ. Wave particle interactions transfer energy between circularly polarized plasma waves and charged particles gyrating around the planetary magnetic field lines. Waves can diffuse electrons not only in pitch angle (a change in the particle’s velocity vector with respect to the local magnetic field) but also in energy resulting in particle acceleration^5^ and an increase in the electron flux at higher energies. The acceleration process depends on the wave type, initial particle energy, pitch angle distribution and the plasma and magnetic field conditions^13^.

Previous attempts to assess electron acceleration by wave particle interactions at Saturn have assumed that whistler mode chorus waves would be most likely to produce acceleration as these waves are very effective at the Earth and Jupiter. However, at Saturn a combination of lower chorus wave power and higher plasma density means that electron acceleration by chorus waves is very weak^11^. We suggest that a different approach is required at Saturn where magnetic and plasma conditions are such that Z-mode waves are intense inside 4 R_S_^14^. Initial studies of Z-mode interactions with electrons at the Earth show they are a very good candidate for electron acceleration^7,15^.

We find that Z-mode waves are very effective at accelerating electrons inside 4 R_S_ and are capable of increasing the electron flux by four orders of magnitude in a year from an essentially empty radiation belt to a value comparable with observations. In comparison to radial diffusion in this region the Z-mode waves are more effective at filling in an empty radiation belt. We also show that Z-mode waves propagate much closer to the magnetic field than previously assumed^16^ and that O-mode waves are much less effective at accelerating electrons than the Z-mode.

## Results

### Z-mode wave observations at Saturn

Saturn’s magnetosphere is dominated by the presence of the Enceladus torus, a region of water group particles emanating from the moon Enceladus just inside 4 R_S_. The plasma density drops off very rapidly with latitude and also decreases notably away from the plasma source of Enceladus. Z-mode waves are frequently observed inside 4 R_S_ where the combination of low plasma density and higher magnetic field strength resulting from proximity to the planet allows an abundance of Z-mode waves to propagate^14^. A case study of Z-mode waves at Saturn^16^ assumed that the direction of wave propagation with respect to the magnetic field (known as the wave normal angle (WNA)) was highly oblique. However, Z-mode waves can also propagate along the magnetic field direction^17,18^.

**Fig. 1:**
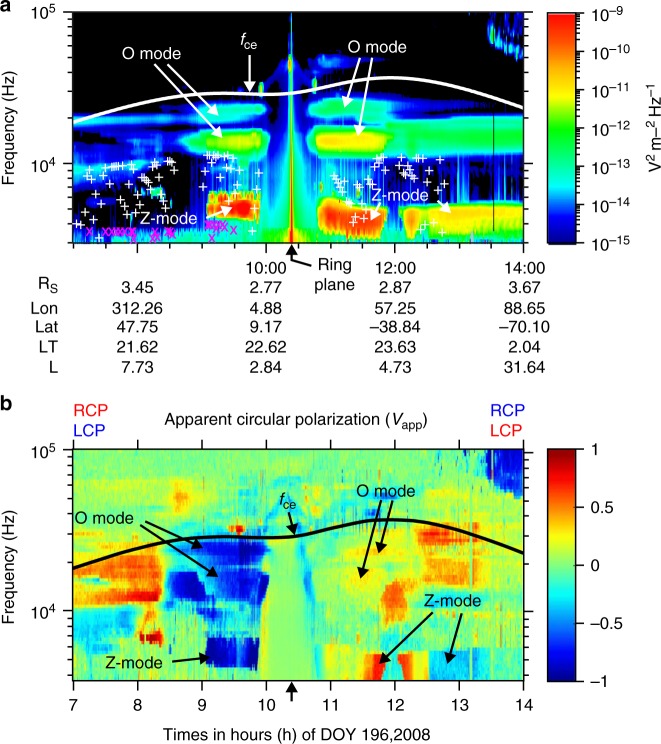
Z-mode and O-mode emission observed by the Cassini RPWS instrument. **a** Electric field power spectral density from RPWS on Cassini. The electron gyro frequency, *f*_ce_, is marked by the solid white line. The white plus signs indicate the plasma frequency and the pink crosses mark the L mode cut-off frequency. **b** Apparent circular polarization^20,21^ as observed by the RPWS for the same time period. Prior to 10:23, apparent circular polarization <0 (blue) is left-hand polarized; after this time >0 (red) is left-hand polarized as indicated by the labels RCP and LCP in the figure

Figure 1a shows an example of wave activity from the Cassini radio and plasma wave instrument^19^ (RPWS) as the spacecraft crossed from the northern to the southern hemisphere. The strong band of emissions near 5 kHz just before 10:00 occurred below the local plasma frequency *f*_pe_ (white+) but above the L mode cut-off frequency *f*_lcut_ (pink x) in a region where *f*_pe_ < *f*_ce_ (gyrofrequency). The apparent polarization^20,21^ of these emissions was predominantly less than zero (Fig. 1b) indicating left-hand circular polarized waves in the plasma convention. Since only Z-mode waves can propagate with left-hand polarization between *f*_lcut_ and *f*_pe_ the emissions have been identified as Z-mode. The emissions at higher frequencies of 10−20 kHz are also left-hand polarized but since in this instance they lie in the range *f*_pe_ < *f* < *f*_ce_ they correspond to left-hand polarized O-mode waves. Z-mode and O-mode waves were also detected after the spacecraft crossed the ring plane in the southern hemisphere, where in this hemisphere an apparent polarization > 0 (red) corresponds to left-hand polarized waves. The 5 kHz wave band remains Z-mode despite changing polarization at ~12:30 because *f*_pe_ falls rapidly at this time such that the 5 kHz band is now in the regime *f*_pe_ < *f* < *f*_ce_, where Z-mode waves are RH polarized. The intensity of the Z-mode waves in Fig. 1a is higher than the O-mode.

**Fig. 2:**
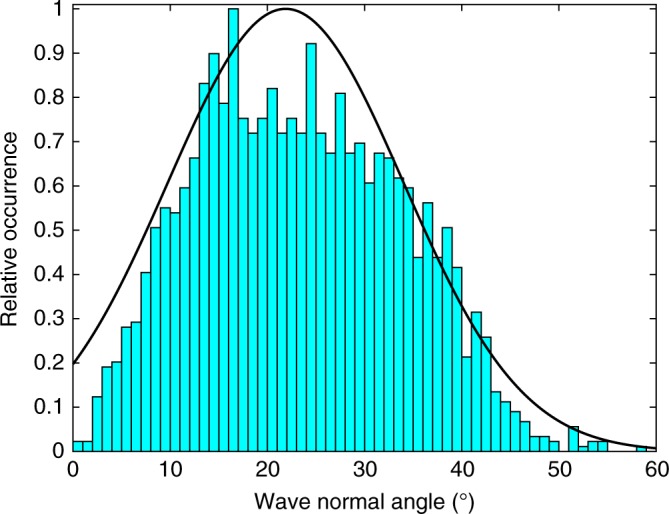
Distribution of wave normal angle for Z-mode waves. These calculations assume a cold plasma index of refraction and are calculated only where density values are available for the data in Fig. 1. The index of refraction is based on the observed value of circular polarization, *f*_pe_ and *f*_ce_. Calculations were done where the apparent circular polarization Stokes parameter, *V*, is >0.5; below this value the calculation is not reliable. The data is shown in blue as number of occurrences normalized to the maximum number of occurrences. The thick black line shows a Gaussian fit to the data with a peak power at a wave normal angle of 21.88°, a width of 17.18°

The Cassini RPWS instrument cannot directly observe the propagation angle of the waves with respect to the local magnetic field for waves with frequencies greater than 2.5 kHz but we can infer this from the polarization of the waves. A circularly polarized wave propagating parallel to the magnetic field with a small WNA will appear with a circular polarization, one propagating across the magnetic field or along the field with a highly oblique WNA will appear with a linear polarization. Fig. 1b shows that there is a mixture of strong and weak left-hand circular polarization with components of linear polarization (not shown) indicating that there is a distribution of WNAs which includes parallel propagation. Instability analysis shows that Z-mode waves can be generated in the field-aligned direction by an unstable distribution of electrons^17,18^. An analysis of the polarization data for this case shows that the WNAs are mostly close to the magnetic field with a peak at ~22° (Fig. 2).

### The effect of the waves on electron energy

**Fig. 3:**
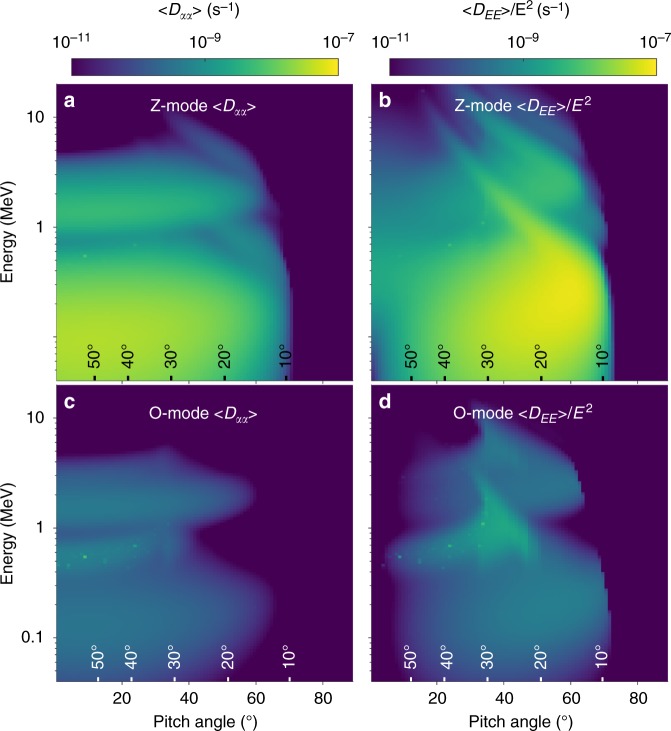
Bounce and drift averaged electron diffusion coefficients. **a** Pitch angle diffusion from Z-mode waves, 〈*D*_*αα*_〉. **b** Energy diffusion from Z-mode waves, 〈*D*_EE_〉/*E*^2^. **c** same as **a** but for O-mode waves. **d** Same as **b** but for O-Mode waves. The color scale indicates the strength of the diffusion. The labels above the horizontal axes show the maximum latitude (mirror latitude) an electron with the corresponding pitch angle would reach during its bounce motion

To model the effect of Z-mode and O-mode waves on the flux of energetic electrons we calculated the diffusion coefficients for the waves using the pitch angle and energy diffusion of ions and electrons (PADIE) code^15^ (described in the Methods section). We have calculated these coefficients for both Z-mode and O-mode waves since these waves often coexist with frequencies near 5 and 20 kHz. Figure 3 shows that electron pitch angle diffusion by Z-mode waves (Fig. 3a) using the WNA distribution from Fig. 2 is much higher than that for O mode waves (Fig. 3c). This is also true for energy diffusion (Fig. 3a and d). Pitch angle diffusion extends up to about 60° (Fig. 3a), indicating that Z-mode waves can scatter electrons into the loss cone at small pitch angles and cause electron loss into the atmosphere of Saturn. Note that the diffusion occurs in two energy bands, one extending up to about 1 MeV and another centered on 1–2 MeV. These bands correspond to Z-mode waves in two frequency bands near 20 and 5 kHz, respectively.

Figure 3b shows that energy diffusion extends from a few tens of keV up to several MeV. Since electron phase space density falls rapidly with increasing energy, this suggests that electron energy diffusion to higher energies, i.e., acceleration, is very effective.

### The local effect of Z-mode electron acceleration

**Fig. 4:**
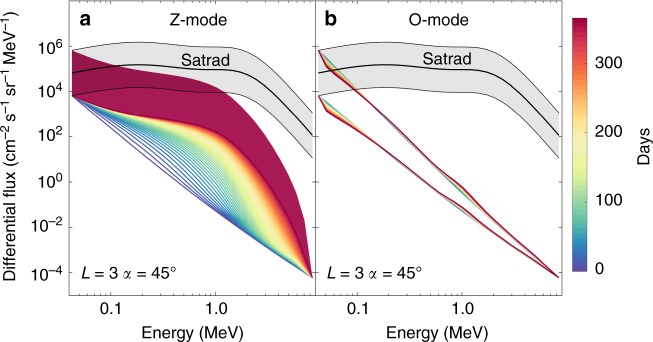
Local electron acceleration due to Z-mode and O-mode waves with no radial diffusion included. The differential electron flux at an L-shell of 3 and pitch angle of 45° is shown evolving from the initial condition (darker blue line) through yellow to finally red lines (lines are separated by 7 days). The broad red band shows the range of possible fluxes after 365 days due to uncertainties in the starting condition. For comparison the SATRAD empirical model of electron flux is shown as the black line with a region of uncertainty shown in gray in each panel. **a** Z-mode waves. **b** O-mode waves

The effect these diffusion coefficients have on the evolution of electron flux at different energies can be simulated using the British Antarctic Survey (BAS) Radiation Belt model^22^ adapted for Saturn (this solves the Fokker–Planck equation as described in the Methods section). Figure 4 shows how the electron energy spectrum evolves over a period of 365 days for the Z-mode and O-mode waves.

Figure 4a shows that Z-mode waves with a small WNA can accelerate electrons significantly from <0.1 to >5 MeV. The energy spectrum reaches the range of the empirical SATRAD model based on Pioneer 11 and Voyager 1 and 2 data after 365 days. It is remarkable that the spectrum can be reproduced over such a wide range of energies from wave acceleration alone with no transport effects included. In comparison, the effect of O-mode waves is very small (Fig. 4b).

The intensity of the waves is the main consideration for the strength of electron acceleration in this region because the plasma density is so low. The 20 kHz Z-mode waves are particularly important in accelerating electrons in the hundreds of keV range in these simulations where the initial condition is essentially an empty radiation belt with a low-energy seed population. To a good approximation in this case an increase or reduction in the overall Z-mode power will change the rate of acceleration by the same amount, e.g. increasing the wave power by a factor of 10 will produce the same level of flux in the simulation 10 times sooner. Cosmic Ray Albedo Neutron Decay (CRAND) will provide an additional source of electrons in the same energy range as the 20 kHz band resonates as will radial diffusion of electrons.

### Z-mode wave acceleration and radial diffusion

**Fig. 5:**
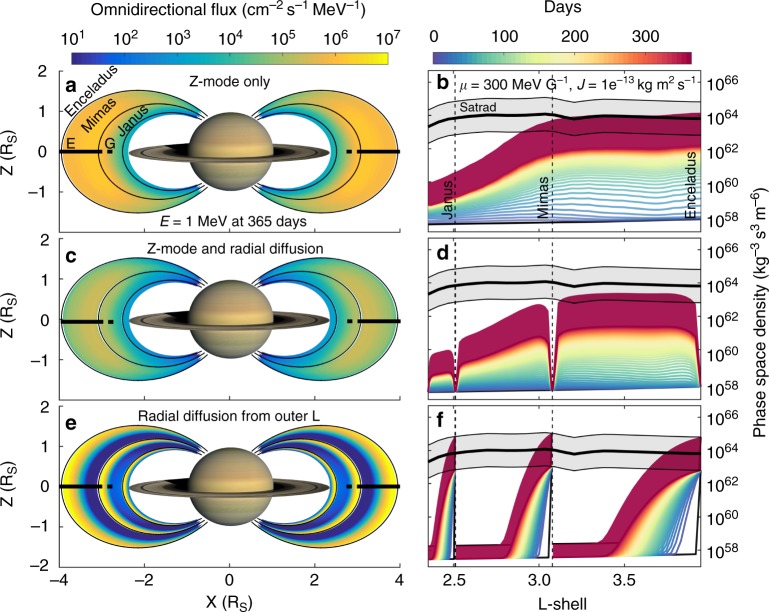
Comparison of the effects of Z-mode waves and radial diffusion inside 4 R_S_. **a**, **c**, **e** Differential flux at 1 MeV after 365 days summed over all pitch angles where the labels E and G refer to the E and G rings. **b**, **d**, **f** Phase space density at constant first and second adiabatic invariants (*µ*,*J*) corresponding to 1 MeV and 50° pitch angle at *L* = 3. SATRAD model and uncertainty range shown in gray, evolution over equal time steps of 7 days starting in black lines through blue and yellow, finishing in red. The broad red band shows the range of possible finishing points for the phase space density after 365 days given uncertainties in the SATRAD model. **a**, **b** Purely local wave acceleration at each L-shell with no radial diffusion. **c**, **d** Local and radial diffusion run together showing the smoothing out of the locally accelerated electrons. **e**, **f** Radial diffusion only using the same initial condition as **a**–**d** but with an added SATRAD energy spectrum just inside the orbit of each moon to form the radial seed population. A worst case absorption has been assumed for the moons, where all electrons are absorbed, in reality the effects of the moons will be smaller

The intensity of the Z-mode waves varies with radial distance from Saturn, exponentially increasing towards the planet^14^. Figure 5a, b show how the strength of the acceleration by Z-mode waves varies with radial distance. The changes in plasma density and magnetic field strength parameters counteract the increase in wave strength as we move closer to the planet^14^ by shifting upwards the energy range over which electrons resonate with the Z-mode waves. Inside ~2.7 R_S_ the acceleration becomes slower, although it is still present all the way to the outer edge of the A-ring at 2.3 R_s_. The A-ring absorbs all energetic particles which is reflected in the zero increase in 1 MeV electrons over time at *L* = 2.3.

Radial diffusion is sufficiently slow that it would take some time longer to fill the region inside 2.7 R_S_ by transporting electrons inwards as shown in Fig. 5c, d where the rate of filling inside 2.7 R_S_ is not increased by adding radial diffusion. Since it is not clear how many electrons can be transported past the orbit of Mimas^23^ or the other moons in this region we have used a worst case scenario and assumed that all electrons will be absorbed (in reality, at the very least, electrons drifting with the same period as the moon orbit will not be absorbed). Fig. 5e, f show a simulation of the effect of radial diffusion on its own over the same period as Fig. 5a–d. This shows that the Z-mode waves are more effective at filling up an empty radiation belt than just radial diffusion.

## Discussion

Our results show that Z-mode waves play a major role in the formation of Saturn’s radiation belts inside the orbit of Enceladus. We suggest the localized nature of this acceleration could explain the observed asymmetries in the high-energy electron population in this region^24^ as asymmetries in Z-mode wave intensity are also observed^14^. Our results suggest that Z-mode waves may also play an important role in radiation belt dynamics at Jupiter, particularly inside the orbit of Io where the plasma density is low, and also at the other magnetized planets of the solar system.

## Methods

### Cassini data

The values for the gyro frequency have been determined from magnetic data taken by the Cassini MAG instrument. The plasma frequency is calculated from data taken by the Langmuir probe on the RPWS instrument on Cassini.

### Radiation Belt Model

The British Antarctic Survey Radiation Belt Model (BAS RBM) uses the well-established method of quasi-linear theory to solve the modified Fokker Planck equation (Eq. (1)) on a planetary scale^22^.

1 $${{\frac{{\partial f}}{{\partial t}} = \frac{1}{{g(\alpha )}}\left. {\frac{\partial }{{\partial \alpha }}} \right|_{E,L}\left( {g(\alpha )D_{\alpha \alpha }\left. {\frac{{\partial f}}{{\partial \alpha }}} \right|_{E,L}} \right) + \frac{1}{{A(E)}}\left. {\frac{\partial }{{\partial E}}} \right|_{\alpha ,L}\left( {A\left( E \right)D_{EE}\left. {\frac{{\partial f}}{{\partial E}}} \right|_{\alpha ,L}} \right) + L^2\left. {\frac{\partial }{{\partial L}}} \right|_{\mu ,J}\left( {\frac{1}{{L^2}}D_{LL}\left. {\frac{{\partial f}}{{\partial L}}} \right|_{\mu ,J}} \right) - \frac{f}{\tau }}}$$

2 $$g\left( \alpha \right) = {\mathrm{sin}}2\alpha \left( {1.3802 - 0.3198\left( {{\mathrm{sin}}\alpha + \sqrt {{\mathrm{sin}}\alpha } } \right)} \right)$$

3 $$A(E) = (E + E_0)\sqrt {(E(E + 2E_0))}$$

where *f* is the phase space density, *t* is time, *α* is the equatorial pitch angle, *D*_*αα*_, *D*_*EE*_, and *D*_*LL*_ are the drift-averaged and bounce-averaged pitch angle, energy, and radial diffusion coefficients, respectively, *E* is the energy, *L* is the L-shell, *µ* and *J* are the first and second adiabatic invariants, *E*_0_ is the electron rest energy, and *τ* is the loss timescale.

The diffusion coefficients are calculated using the PADIE code^15^ which solves the resonance condition for cold plasma dispersion in a magnetic field. We use a centered dipole magnetic field with an equatorial surface magnetic field strength, *B*_0_, of 2.1951 × 10^−5^ T which is a good approximation this close to Saturn. We also use a model of the plasma density^25^ including the density from the rings based on the Saturn Orbit Insertion data from Cassini.

The BAS RBM uses implicit methods to solve Eq. (1) on a two-grid system with one grid in *α, E*, and *L* and the other in *µ, J*, and *L* (*L* is the same in both grids). The pitch angle and energy diffusion are calculated as a separate step^26^ using a time step of 5 s and then interpolated using cubic splines onto the *µ, J, L* grid which is solved every 500 s. The grid resolution is 90 × 30 × 30 points (*α, E, L*) and the energy grid uses equal spacing in the natural log of the energy (from 40 to 8000 keV at the maximum *L* value for the simulations in Fig. 4, from 40 to 3900 keV in Fig. 5 also at maximum *L*). We calculate the pitch angle at which all electrons are assumed to be lost to the atmosphere (the loss cone angle) at an altitude of 1000 km above the planet radius of 60,330 km.

The boundary conditions make use of the SATRAD^27^ electron distribution model at Saturn. This is code that encapsulates the data from Pioneer 11, and Voyagers 1 and 2 and is freely available to download with documentation at http://www.openchannelfoundation.org/projects/SATRAD/. We use the SATRAD differential flux value to define the minimum energy boundary. Although the SATRAD model is the best currently available for this region at Saturn there are significant uncertainties due to the small amount of data included. Comparing the SATRAD model to the spacecraft passes that it is derived from using figure A1 in the SATRAD report shows that there is at least an order of magnitude uncertainty in the fluxes from the model at energies of a few hundred to a few MeV. We therefore show a range of fluxes that span from SATRAD divided by 10 to SATRAD multiplied by 10.

The maximum energy boundary is set at a low value of phase space density, *f* = 10^50^ kg^−3^ s^3^ m^−6^ at the minimum pitch angle. The inner and outer L-shell boundaries required for the runs involving radial diffusion are subject to the condition *f* = 0 with the inner boundary set just outside each moon orbit and the outer boundary just inside (three separate runs are used to cover the L space from Epimetheus to Enceladus, separated by each moon). The maximum and minimum boundaries in *α* are set to *∂f*/*∂α* = 0. The initial grid is set to be a straight line in log_10_(*f*) vs. log_10_(*E*) with a negative gradient from *E*_min_ to *E*_max_. The precise gradient of the initial grid is unimportant^10^ since the initial rise from a step function of *f* with *E* at *E*_min_ is so rapid compared to the overall timescale. For the radial diffusion only run a seed population at maximum *L* (just inside each moons orbit) is added by including the flux spectrum from the SATRAD model. The initial dependence of *f* on *α* is set at (sin *α*)^2^ to match the dependence in SATRAD.

The uncertainty in the SATRAD values also has an important impact on the initial and boundary conditions for the model runs with the flux at minimum energy acting as a source. We have therefore shown the range of possible fluxes that could be reached assuming that the flux and the minimum energy boundary varies within the SATRAD uncertainty range.

We have deliberately included a worst case scenario of moon absorption to demonstrate that the moons do not have a significant impact on the wave acceleration even when combined with radial diffusion. In fact the moons have a much less significant effect on the electrons in this region than they do on the protons^24^. Also some moons are more effective absorbers of energetic electrons than others^23^. To further isolate the effects of the Z-mode and O-mode waves we have not included CRAND as a source, or losses due to rings and other plasma waves.

### Diffusion coefficients

The pitch angle diffusion coefficient, *D*_*αα*_, is the sum of the coefficients from the wave–particle interactions and the diffusion introduced by collisions in Saturn’s atmosphere and the neutral torus produced by Enceladus. The PADIE^15^ code is used to calculate *D*_*αα_*wave_ with the information on the wave properties from the Z-mode wave survey^14^. Z-mode waves are assumed to exist in two wave bands, 5 and 20 kHz, with wave power assumed to be a Gaussian shape with frequency. In general the 20 kHz banded emission can be either O-mode, as in Fig. 1, or Z-mode as in the case presented by Gu and co-authors^16^ depending on the plasma properties. The frequency values of the 5 kHz band are peak 5.0 kHz, bandwidth 1.1 kHz, lower cut-off 3.5 kHz, and upper cut-off 7.5 kHz^14^. For the 20 kHz band these are: peak 17.0 kHz, bandwidth 3.0 kHz, lower and upper cut-offs, 10.0 and 26.0  kHz^16^.

The wave power^14^ is also assumed to vary with both latitude, *λ*, and radial distance, *R* (Supplementary Figure 1). We have assumed that *R* is radial distance along the equator and therefore equivalent to *L* in a dipole field.

4 $$B_{\mathrm {W}}^2 = \frac{{1.75 \times 10^{ - 4}}}{{R^{5.6}}}{\mathrm {e}}^{ - \left( {\frac{{\lambda - \lambda _{\mathrm {m}}}}{{\lambda _{\mathrm {w}}}}} \right)^2}$$

The latitude of peak power is *λ*_m_ = 25.0° and the width is *λ*_w_ = 14.142°. Note that wave power taken from Figure 7d in the paper by Menietti and co-authors^14^ is the average wave power over latitude but the power required in the equation above is the peak power with latitude. We therefore divide the average wave power by the mean of a Gaussian with a peak power of 1 with the peak power latitude and width values given above, over the range of 0–50° latitude (which is the range over which data is available). This results in the factor of 1.75 × 10^−4^ in the equation above.

The WNA of the waves is also assumed to have a Gaussian shape based on the information in Fig. 2 with waves having a peak power at a WNA of 21.88°, a width of 17.18° and lower and upper cut-offs of 0° and 56.24°.

The O-mode at Saturn tends to appear in the same narrow band structures as the Z-mode. There are no published surveys of the O-mode at Saturn so for the best comparison of the acceleration capabilities of the two wave modes we replicate all of the Z-mode wave parameters for the O-mode.

For the PADIE calculations we use the cold plasma density model for the Enceladus torus^25^ combined with the ring plasma density from Cassini Saturn Orbit Insertion data^25^. We use a scale height^28^ to extend the equatorial densities to higher latitudes. We use a centered dipole magnetic field model as in the BAS RBM. One further consideration when calculating the diffusion coefficients for the Z-mode waves is the presence of a reverse resonance cone which exists where the wave frequency is lower than the upper hybrid frequency but higher than both the electron gyro frequency and plasma frequency.

We have included scattering from particles in the neutral torus created by Enceladus by calculating a value of pitch angle scattering for the presence of water ions. We use a model of water ions from Enceladus^29^ to calculate *D*_*αα_*torus_^30^. We have assumed that the neutral torus is azimuthally symmetric around Saturn and consists of H_2_O, OH, and O with a defined scale height away from the equator^31^.

We have included the pitch angle scattering due to the atmosphere of Saturn using the same method^30^ to calculate *D*_*αα_*atmos_. This gives a very high value for the total *D*_*αα*_ at pitch angles up to the loss cone angle. To avoid cubic spline interpolation overshoot issues near the loss cone we use the value of *D*_*αα_*atmos_ at *α* = 0° and then we use a quadratic curve to join this up to the value of *D*_*αα_*atmos_ at the pitch angle grid point closest to the loss cone. The loss timescale within the loss cone is based on the time taken for the electron energy to decay^32^ by 1/*e*. The ratio of *D*_*αα_*atmos_ to the loss time is calculated at *α* = 0° and then the loss time is calculated at other points inside the loss cone using this ratio and the *D*_*αα_*atmos_ at each grid point.

The radial diffusion coefficient *D*_*LL*_ = 2 × 10^−14^ L^7^ s^−1^ is calculated from Cassini electron data^2^. We regard this as an upper limit for the radial diffusion as the value is likely to be contaminated by wave–particle interactions and to some extent the CRAND process.

## Electronic supplementary material

Supplementary Information

## Data Availability

Cassini RPWS and MAG data are archived in calibrated, full resolution at the NASA Planetary Data System website: http://pds.nasa.gov. The Meudon Cassini RPWS/HFR polarization database is located at http://www.lesia.obspm.fr/kronos/data. Other datasets generated during the current study are available from the corresponding author on reasonable request.

## References

[1] Kellogg PJ (1959). Van Allen radiation of solar origin. Nature.

[2] Kollmann P (2011). Energetic particle phase space densities at Saturn: Cassini observations and interpretations. J. Geophys. Res..

[3] Simpson JA, Bastian TS, Chenette DL, McKibben RB, Pyle KR (1980). The trapped radiations of Saturn and their absorption by satellites and rings. J. Geophys. Res..

[4] Walt, M. *Introduction to Geomagnetically Trapped Radiation* (Cambridge University Press, New York, USA, 1994).

[5] Horne RB (2005). Wave acceleration of electrons in the Van Allen radiation belts. Nature.

[6] *Waves, Particles and Storms in Geospace,* 1st edn (Oxford University Press, New York, USA, 2016).

[7] Xiao FL, Zhang S, Su ZP, He ZG, Tang LJ (2012). Rapid acceleration of radiation belt energetic electrons by Z-mode waves. Geophys. Res. Lett..

[8] Millan RM, Baker DN (2012). Acceleration of particles to high energies in earth’s radiation belts. Space Sci. Rev..

[9] Horne RB (2008). Gyro-resonant electron acceleration at Jupiter. Nat. Phys..

[10] Woodfield EE, Horne RB, Glauert SA, Menietti JD, Shprits YY (2014). The origin of Jupiter’s outer radiation belt. J. Geophys. Res..

[11] Shprits YY, Menietti JD, Gu X, Kim KC, Horne RB (2012). Gyroresonant interactions between the radiation belt electrons and whistler mode chorus waves in the radiation environments of Earth, Jupiter, and Saturn: a comparative study. J. Geophys. Res..

[12] Lorenzato L, Sicard A, Bourdarie S (2012). A physical model for electron radiation belts of Saturn. J. Geophys. Res..

[13] Lyons LR (1974). General relations for resonant particle diffusion in pitch angle and energy. J. Plasma Phys..

[14] Menietti JD (2015). Survey of Saturn Z-mode emission. J. Geophys. Res..

[15] Glauert SA, Horne RB (2005). Calculation of pitch angle and energy diffusion coefficients with the PADIE code. J. Geophys. Res..

[16] Gu Xudong, Thorne Richard M., Ni Binbin, Ye Sheng-Yi (2013). Resonant diffusion of energetic electrons by narrowbandZmode waves in Saturn's inner magnetosphere. Geophysical Research Letters.

[17] Wu CS, Yoon PH, Freund HP (1989). A theory of electron-cyclotron waves generated along auroral field lines observed by ground facilities. Geophys. Res. Lett..

[18] Menietti JD (2016). Source region and growth analysis of narrowband Z-mode emission at Saturn. J. Geophys. Res..

[19] Gurnett DA (2004). The Cassini radio and plasma wave investigation. Space Sci. Rev..

[20] Cecconi B, Zarka P (2005). Direction finding and antenna calibration through analytical inversion of radio measurements performed using a system of two or three electric dipole antennas on a three-axis stabilized spacecraft. Radio Sci..

[21] Fischer G (2009). Elliptical polarization of Saturn kilometric radiation observed from high latitudes. J. Geophys. Res..

[22] Glauert SA, Horne RB, Meredith NP (2014). Three dimensional electron radiation belt simulations using the BAS Radiation Belt Model with new diffusion models for chorus, plasmaspheric hiss and lightning-generated whistlers. J. Geophys. Res..

[23] Roussos E (2007). Electron microdiffusion in the Saturnian radiation belts: Cassini MIMI/LEMMS observations of energetic electron absorption by the icy moons. J. Geophys. Res..

[24] Paranicas C (2010). Asymmetries in Saturn’s radiation belts. J. Geophys. Res..

[25] Persoon AM, Gurnett DA, Kurth WS, Groene JB, Faden JB (2015). Evidence for a seasonally dependent ring plasma in the region between Saturn’s A Ring and Enceladus’ orbit. J. Geophys. Res..

[26] Strang G (1968). On the construction and comparison of difference schemes. SIAM J. Numer. Anal..

[27] Garrett HB, Ratliff JM, Evans RW (2005). Saturn radiation (SATRAD) model. JPL Publ..

[28] Persoon AM (2013). The plasma density distribution in the inner region of Saturn’s magnetosphere. J. Geophys. Res..

[29] Cassidy TA, Johnson RE (2010). Collisional spreading of Enceladus’ neutral cloud. Icarus.

[30] Abel B, Thorne RM (1998). Electron scattering loss in Earth’s inner magnetosphere: 1. Dominant physical processes. J. Geophys. Res..

[31] Persoon AM, Gurnett DA, Kurth WS, Groene JB (2006). A simple scale height model of the electron density in Saturn’s plasma disk. Geophys. Res. Lett..

[32] Spjeldvik WN, Thorne RM (1975). Cause of storm after effects in middle latitude D-region. J. Atmos. Terr. Phys..

