# Supplementary material for: Formation of electron radiation belts at Saturn by Z-mode wave acceleration
Source: Nat Commun. 2018 Nov 29;9:5062. doi: 10.1038/s41467-018-07549-4 (PMC6265320; doi:10.1038/s41467-018-07549-4)
Supplement: Supplementary file 1 — Supplementary Information [file 41467_2018_7549_MOESM1_ESM.pdf]

Supplementary Information

Formation of electron radiation belts at Saturn by Z-mode wave acceleration

Woodfield et al.

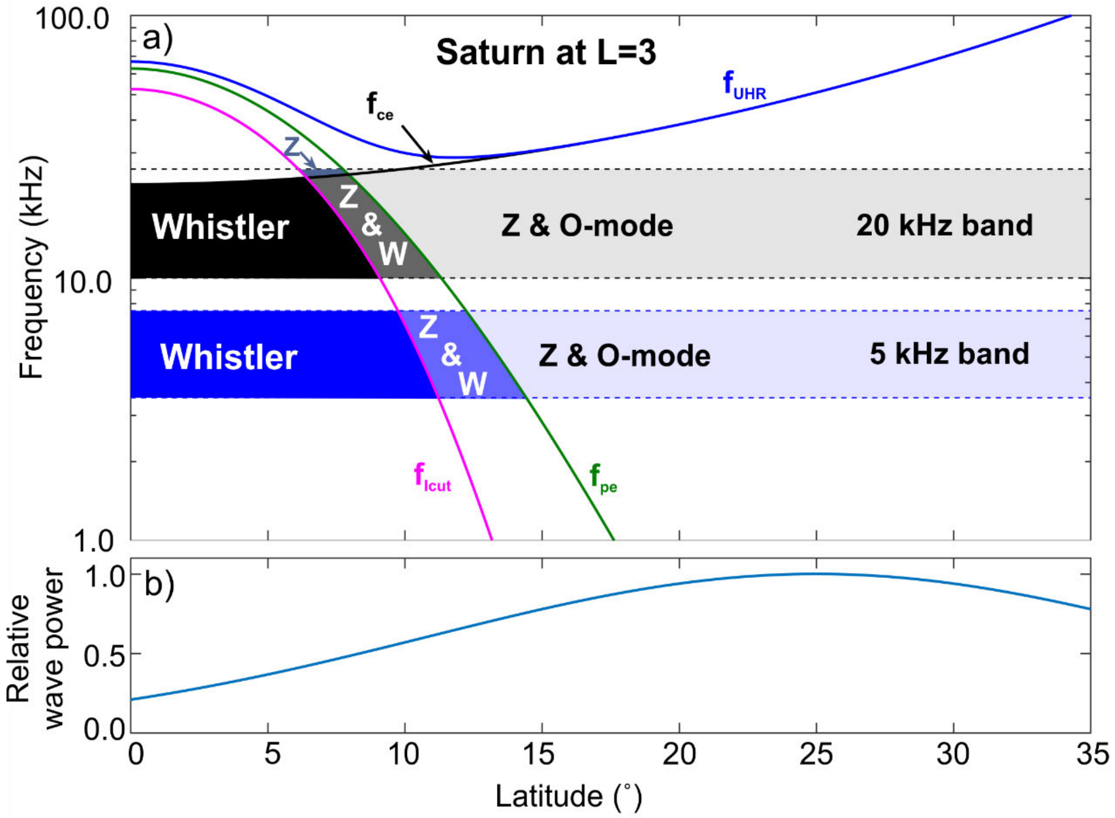

Supplementary Figure 1 The effect of magnetic latitude on wave propagation. **a** The characteristic frequencies at Saturn at L=3, f_{ce} is the electron gyro frequency, f_{pe} is the plasma frequency, f_{UHR} is the upper hybrid resonance frequency and f_{lcut} is the L mode cut-off frequency. In the two frequency bands used in the calculations (indicated by the coloured areas between the dashed lines) the wave types which can propagate change with latitude. The characteristic frequencies, f_{ce}, f_{pe}, f_{UHR}, f_{lcut}, change with L-shell but the Z-mode frequency bands do not. **b** Normalized wave power used in the diffusion coefficient calculations versus latitude, this does not change with L-shell.
